# Circulating tumor cells as biomarkers in melanoma: techniques, challenges, and clinical applications

**DOI:** 10.3389/fcell.2026.1857720

**Published:** 2026-07-29

**Authors:** Elisabetta Broseghini, Giorgio Durante, Alessia Soru, Francesca Bianchi, Francesca Comito, Emi Dika, Manuela Ferracin, Giulia Gallerani

**Affiliations:** 1 IRCCS Azienda Ospedaliero-Universitaria di Bologna, Bologna, Italy; 2 Department of Medical and Surgical Sciences (DIMEC), University of Bologna, Bologna, Italy

**Keywords:** biomarkers, CTC, cutaneous melanoma, liquid biopsy, prognosis

## Abstract

Cutaneous melanoma is a malignant tumor and remains a clinical challenge due to unmet needs in risk stratification, real-time treatment monitoring, and early detection of progression or resistance. Early detection is crucial, as prognosis significantly worsens once metastasis occurs. Liquid biopsy has emerged as a powerful, minimally invasive tool for early diagnosis, real-time disease monitoring, and therapeutic guidance. Among its components, circulating tumor cells (CTCs) are rare yet clinically informative, providing insights into tumor heterogeneity, metastatic potential, and prognosis. Given its high heterogeneity, melanoma could particularly benefit from CTC analysis to inform personalized treatment strategies and improve clinical outcomes. Quantification of melanoma CTCs remains limited by low abundance and heterogeneity, as well as the lack of standardized isolation methods across laboratories. This review summarizes current methods for melanoma CTC detection and isolation. It also explores the clinical significance of melanoma CTCs, addressing the challenges and future opportunities for their implementation in clinical practice.

## 1 Introduction

Cutaneous melanoma is a malignant tumor that accounts for 1.4% of all cancer-related deaths and represents the fifth most common cancer diagnosis in the US, comprising 5.1% of all cancer diagnoses, according to the latest SEER data (SEER, 2025). Melanoma risk depends on a combination of environmental factors (Rastogi et al., 2010), genetic alterations (Toussi et al., 2020; Broseghini et al., 2025a; Broseghini et al., 2021; Durante et al., 2021), and phenotypic traits (Arisi et al., 2018). With the continuous rise in melanoma incidence, guidelines for its diagnosis, staging, and treatment are regularly revised and updated (Broseghini et al., 2024).

The diagnosis of melanoma, which is primarily based on dermoscopy and histopathological examination, is challenging because of its variable presentation and the subjectivity of interpretation. Melanoma diagnosis shows substantial interrater variability even among expert pathologists, with complete agreement in just over half of cases and pronounced discordance for non-invasive lesions, underscoring the diagnostic challenges of early-stage melanoma and the need for more robust consensus approaches in routine practice (Haggenmuller et al., 2025). After clinical diagnosis, histopathology assessment is essential for determining prognosis. In early-stage melanoma (stages I–II), key prognostic features include Breslow thickness, ulceration, mitotic rate, and tumor-infiltrating lymphocytes (TILs) (Dika et al., 2020; Scolyer et al., 2020). Sentinel lymph node biopsy (SLNB) is used to evaluate potential metastasis, with melanocytic markers such as S-100, Melan-A, HMB45, and SOX10 aiding confirmation (Szumera-Cieckiewicz et al., 2020). In stage III disease, survival correlates with the number of metastatic lymph nodes (Zettersten et al., 2002), while microscopic satellites and in-transit metastases signal an elevated risk of recurrence (Bann et al., 2019). In advanced melanoma (stage IV), high serum LDH levels indicate greater tumor burden and poorer survival (Gray et al., 2014). Additional diagnostic procedures for early-stage melanoma are essential to reduce mortality rates, as melanoma prognosis significantly depends on early detection before metastatic spread (Millet et al., 2017; Strazzulla et al., 2019; Slusher et al., 2024).

In recent years, liquid biopsy has evolved from a research technique into a crucial element of clinical oncology (Palmirotta et al., 2018; Broseghini et al., 2025b). Liquid biopsy enables analysis of tumor biomarkers in bodily fluids (Martins et al., 2021), providing a minimally invasive method for early diagnosis and longitudinal monitoring of disease progression, without the need for direct manipulation of tumor tissue (Martins et al., 2021; Kuligina et al., 2024; Pantel et al., 2019).

Unlike other circulating biomarkers, circulating tumor cells (CTCs) are the only viable analyte that can be physically isolated through liquid biopsy for subsequent downstream analyses (Smit and Pantel, 2024; Cohen et al., 2008; de Bono et al., 2008; Hayes et al., 2006; Rossi et al., 2021). CTCs are malignant cells shed from primary and metastatic tumors into the bloodstream, characterized by the ability to detach from the primary tumor and enter the bloodstream or lymphatic vessels. Only a small fraction of CTCseventually exit the bloodstream at distant tissue and, ultimately adapt, colonize, and proliferate to establish new metastases (Massague and Obenauf, 2016; Munoz-Arcos et al., 2023; Pantel and Speicher, 2016). These CTCs exhibit distinct characteristics, including variable stemness, epithelial-to-mesenchymal transition (EMT), and tumor-initiating capacity, that contribute to their metastatic potential (Janjua et al., 2025; Aceto et al., 2014; Visioli et al., 2019; Yu et al., 2013).

Despite their rarity, often comprising 1 in every 10^5^–10^6^ peripheral blood mononuclear cells (PBMCs), CTCs represent a highly heterogeneous population. CTCs may exhibit epithelial (EpCAM+), mesenchymal (EpCAM^−^/Vimentin^+^, N-cadherin^+^) or hybrid phenotypes, the latter displaying features of both epithelial and mesenchymal states (Gwark et al., 2020; Tellez-Gabriel et al., 2020; Vismara et al., 2020; Pineiro et al., 2020).

A particularly relevant subpopulation is represented by cancer stem cell-like CTCs (CSC-CTCs), characterized by self-renewal capacity and strong tumor proliferation potential, which are thought to be primarily responsible for metastatic colonization and disease relapse (Le et al., 2008; Sun and Qiu, 2013). The origin of CSC-CTCs can be explained by either differentiated cancer cells undergone EMT process or EMT in CSC cells, and regardless of their cell of origin this mechanism could explain the high heterogeneity displayed by CTCs.

Additionally, CTCs can circulate as single cells or as multicellular aggregates known as CTC clusters, which exhibit up to a 100-fold higher metastatic efficiency compared to single CTCs (Aceto et al., 2014; Aceto et al., 2015; Fabisiewicz and Grzybowska, 2017). CTC clusters can be homotypic when formed only by CTCs or heterotypic clusters that incorporate cancer cells and non-cancerous cells such as neutrophils, platelets, myeloid cells, cancer-associated fibroblast (CAFs) (Aceto, 2020).

**FIGURE 1:**
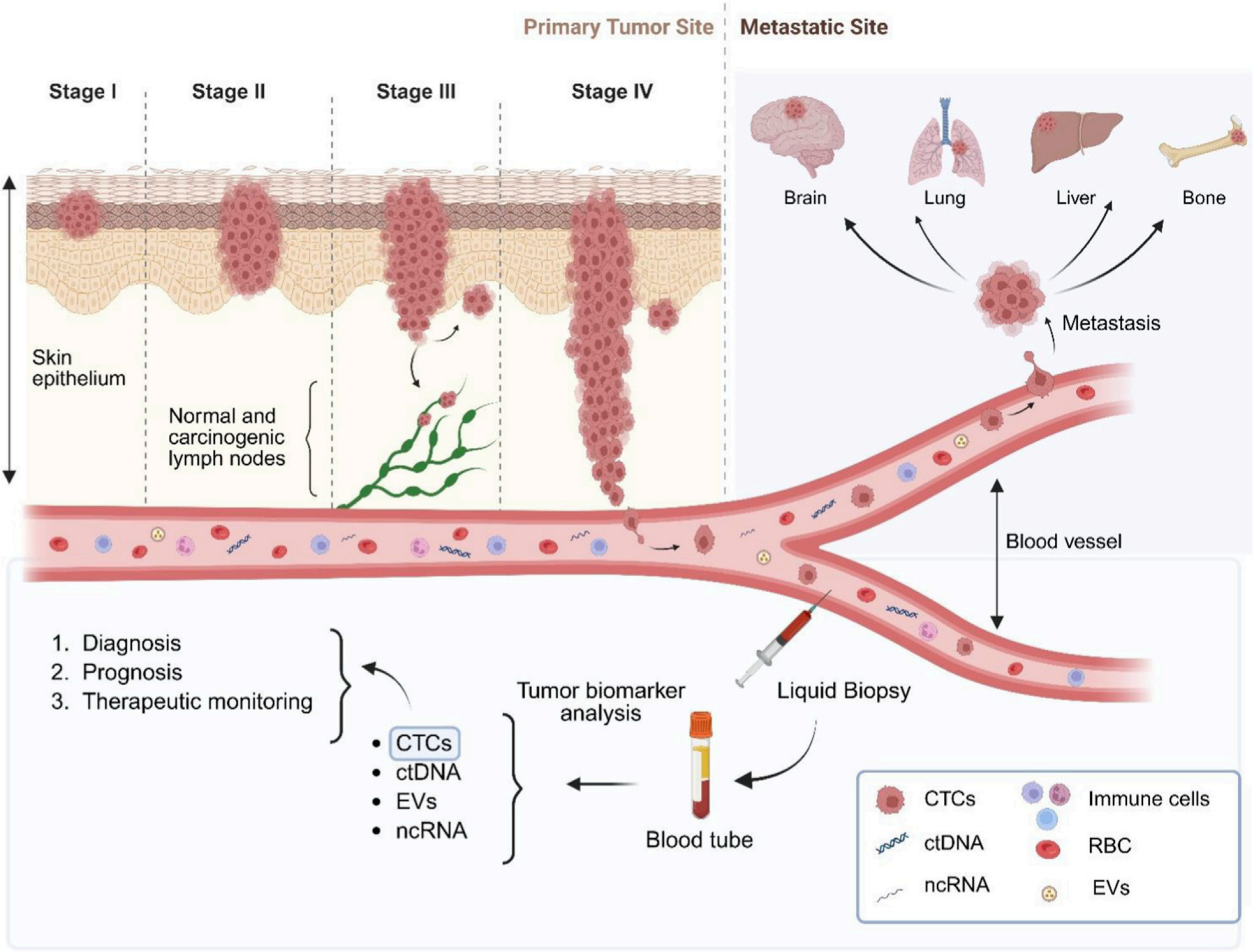
Circulating tumor cells (CTCs) in melanoma progression. This figure illustrates how melanoma tumor cells shed into the bloodstream from the primary tumor, becoming CTCs and eventually seeding distant organs to give rise to metastases. Liquid biopsy provides tumor information by the analysis of CTCs, Circulating tumor DNA (ctDNA), Extracellular vesicles (EVs), and noncoding RNA (ncRNA). In this review, we focused on the use of CTC in melanoma diagnosis, prognostic assessment, and therapeutic monitoring. The figure was created in BioRender. Broseghini, E. (2026) https://BioRender.com/c90jedv.

Although characterized by considerable heterogeneity and rarity, CTCs serve as a clinically relevant tool for studying tumor progression (Gallerani et al., 2021; Rossi et al., 2022; Visal et al., 2022). However, their low frequency poses a challenge for clinical applications, necessitating standardized methods for their isolation and characterization (Rushton et al., 2021). Due to its high degree of heterogeneity, melanoma could significantly benefit from the findings provided by CTC analysis, especially in prognosis and treatment decisions (Ricciardi et al., 2023) (Figure 1).

The role of CTCs in melanoma has been extensively investigated over the years, generating important insights into their biological characteristics and potential clinical applications, as reflected by several reviews published on the subject (Khoja et al., 2013; Girotti et al., 2016; Rapanotti et al., 2024; Huang et al., 2022; Shoji et al., 2022; Marsavela et al., 2018; Huang and Hoon, 2016; Mocellin et al., 2006; Ghossein et al., 2000). However, the field has evolved rapidly in recent years with the development of novel isolation platforms, combined methods, and *in vivo* enrichment strategies. The present review provides an updated and comprehensive synthesis of both the technical landscape and the clinical evidence, with particular attention to the translational gap between research findings and routine clinical applicability. We critically evaluate the readiness of available platforms for clinical translation and discuss the diagnostic, prognostic, and monitoring roles of CTCs within a unified framework.

This manuscript is a narrative review. Relevant literature was identified through searches of PubMed/MEDLINE and Scopus using the terms “circulating tumor cells” AND “melanoma”, combined with “isolation”, “detection”, “diagnosis”, “prognosis”, and “liquid biopsy”, covering publications up to December 2025. No formal systematic protocol was applied. Studies were selected based on their relevance to the topics discussed, prioritizing original research, clinical studies, and review articles in English on CTC detection, isolation, molecular characterization, or clinical application in melanoma patients.

## 2 CTC detection and isolation

The low frequency and heterogeneous nature of CTCs present significant challenges in developing reliable detection methods. Several technologies have been implemented to enrich CTCs based on their physical and biological properties, such as size, density, charge, and marker expression. CTC detection and isolation methods are categorized into label-independent approaches, namely biophysical approaches exploiting CTC’s unique properties, and label-dependent approaches, namely immunocapture techniques targeting specific cell-surface markers such as the epithelial cell adhesion molecule (EpCAM). While EpCAM is widely expressed in most solid tumors it could fail to detect CTCs originating from cancer with low- EpCAM expression levels. Indeed, some adenocarcinomas such as hepatocellular carcinomas, clear cell renal cell cancer, urothelial cancer and squamous cell cancers or breast cancer and neurogenic cancers are low expressing or EpCAM negative (Spizz et al., 2011). Moreover, as previously described, CTC can undergo EMT and acquire mesenchymal or hybrid phenotypes, defined by downregulation of EpCAM expression. Altogether, label-dependent CTC detection, mainly based on EpCAM levels, are affected by this downregulation (Gwark et al., 2020; Tellez-Gabriel et al., 2020; Vismara et al., 2020; Pineiro et al., 2020).

Each strategy captures distinct yet partially overlapping CTC populations. Moreover, there are also combined methods, which use both physical and immunological approaches, and *in vivo* enrichment methods, such as diagnostic leukapheresis and photoacoustic flow cytometry platform (Janjua et al., 2025; Rushton et al., 2021).

### 2.1 Immunocapture methods

Immunocapture methods include technologies based on immunomagnetic positive and negative enrichment, microfluidic immunocapture, and nanomaterial-based enhancement (Table 1).

**TABLE 1:** List of immunocapture methods used for melanoma CTC detection and isolation.

Methods	Platform	Description	Advantages	Disadvantages	Ref.
Positive enrichment	CellSearch System	Based on immunomagnetic enrichment (MCAM, MCSP)	FDA-approved* High sensitivity, specificity, and reproducibility	Low sensitivity for CTCs with low EpCAM expression Based on previously known cell surface markers	Marsavela et al. (2018), Ligthart et al. (2011), Rao et al. (2011)
	Magnetic activated cell sorting (MACS)	Based on immuno-labeled magnetic microbeads coated with melanoma marker antibodies, such as MCSP	High sensitivity Automated separation	Expensive	Miltenyi et al. (1990), Galanzha et al. (2019b)
Negative enrichment	EasySep	Based on negative selection, immunomagnetic isolation with a bispecific tetrameric antibody complex (TAC) against leukocytes	Easy and rapid method Label-free isolation	Low recovery efficiency Low purity levels	Liu et al. (2011), Fusi et al. (2011)
	RosetteSep CTC	Based on immunodensity-negative selection using an antibody cocktail targeting multiple blood cell markers	Easy and quick procedure Isolation of viable CTCs	Low sample purity	Girotti et al. (2016), He et al. (2008)
	CTC-Chip	Based on a microfluidic chip with anti-EpCAM-coated microposts in controlled flow	High sensitivity Isolation of viable CTCs Short processing time	Does not detect EpCAM-negative CTCs	Sequist et al. (2009), Nagrath et al. (2007)
Microfluidic immunocapture	Herringbone (HB) CTC-Chip	Based on a Microfluidic chip with anti-EpCAM-coated microposts in a disrupted-flow system	Higher capture efficiency than CTC-Chip Isolation of viable CTCs	Does not detect EpCAM-negative CTCs	Sharma et al. (2018), Nagrath et al. (2007), Luo et al. (2014), Sarioglu et al. (2015), Stott et al. (2010)
	Nanovelcro Chip	Based on an anti-EpCAM-coated SiNS to immobilize CTCs on the chip	High sensitivity Isolation of viable CTCs Short processing time	Does not detect EpCAM-negative CTCs	Jan et al. (2018), Hou S. et al. (2013)
	IsoFlux System	Based on a microfluidic system with controlled flow and immunomagnetic positive selection	Semi-automated platform High sensitivity	Time-consuming Does not capture EpCAM-negative CTCs	Rushton et al. (2021), Agerbaek et al. (2018), Po et al. (2019)

* for metastatic breast, prostate, and colorectal cancers.

#### 2.1.1 Positive enrichment

A major advancement in CTC isolation has been the development of affinity-based capture methods for cells expressing EpCAM. EpCAM plays a central role in tumor growth, EMT, and metastasis, but its expression is generally restricted to epithelial-derived tumors. Because most solid cancers originate from epithelial cells, a substantial proportion of CTCs continue to express EpCAM on their membranes after spreading through the bloodstream, whereas normal epithelial cells expressing EpCAM are exceedingly rare in peripheral blood (Edd et al., 2022).

The CellSearch (Menarini Silicon Biosystems, Bologna, Italy), first described by Allard et al. (Allard et al., 2004), is the “Gold Standard” platform for CTC isolation and couples immunomagnetic enrichment using ferrofluid coated with anti-EpCAM antibodies with in-device immunostaining for the cancer cell marker cytokeratin (CK) −8, −18, and −19, the leukocyte marker CD45, and nuclear stain 4′,6-diamidino-2-phenylindole (DAPI). It consists of two tools: the Autoprep, for the capture and immunostaining of CTCs, and the CellTracks Analyzer, a semi-automated fluorescence microscope that captures images of individual cells using four fluorescence channels and presents them for operator review and CTC counting. CellSearch defines a cell as CTC when it is CK-positive, CD45-negative, with an intact nucleus (indicated by DAPI staining) and a round or oval shape, with a minimum diameter of 4 µm (Ligthart et al., 2011). CellSearch is one of the Food and Drug Administration (FDA)-approved platforms for CTC enumeration in metastatic breast, prostate, and colorectal cancers, where CTC enumeration has been used as a prognostic tool since it has been associated with overall survival and/or progression-free survival (Hayes et al., 2006; Cristofanilli et al., 2004; Danila et al., 2016; Goldkorn et al., 2024; Sastre et al., 2008).

However, the use of CellSearch targeting EpCAM is not recommended for melanoma due to low sensitivity and inconsistent results, as melanoma is derived from melanocytes, cells that originate from the neural crest, thus not expressing the EpCAM marker (Alix-Panabieres and Pantel, 2014). To overcome this issue, the CELLTRACKS® Circulating Melanoma Cell kit has been developed for isolating melanoma CTCs. This kit captures CTCs by targeting the melanoma cell adhesion molecule (MCAM) and detects them using immunostaining with melanoma-associated chondroitin sulfate proteoglycan (MCSP, also known as CSGP4 and HMW-MAA) (Marsavela et al., 2018). Several studies have applied the CellSearch platform to evaluate CTC counts in patients, providing insights into detection rates and their association with disease progression.

Rao et al. detected 0 to 8042 CTCs per 7.5 mL of blood in 44 patients, with more than 10 CTCs found in only 3 patients (4%) (Rao et al., 2011). Khoja et al. used the melanoma-specific CellSearch kit to detect CTCs in 101 metastatic melanoma patients, finding between 0 and 36 CTCs per 7.5 mL of blood before treatment, with 40% of patients having at least one CTC (Khoja et al., 2013). Lucci et al. found that melanoma CTCs were detectable in 37% of stage III melanoma patients, and the presence of at least one CTC in these patients was independently linked to melanoma relapse (Lucci et al., 2020). Additionally, the same groups found that CTCs appear before imaging-confirmed relapse in stage III melanoma patients (Lucci et al., 2023). Hall et al. determined that the presence of one or more CTCs in an initial sample of stage IV melanoma patients was indicative of disease progression within 180 days (Hall et al., 2018). Freeman et al. found that using antibodies targeting MCSP, MCAM, ATP-binding cassette transporter 5 (ABCB5), and Low-affinity Nerve Growth Factor Receptor (LNGFR, CD271) significantly increased the proportion of metastatic melanoma patients with detected CTCs compared to using MCSP or MCAM alone. This approach revealed the high diversity of melanoma CTCs and improved sensitivity over single-marker methods. In their study, 73.9% of patients from all stages had at least one CTC/mL of blood, with a range of 0–2.5 CTCs/mL. Despite the use of multiple markers to improve sensitivity, the capture efficiency remained as low as 34%, indicating that only a small number of melanoma CTCs were successfully isolated with this multimarker approach (Freeman et al., 2012).

An alternative immunomagnetic positive enrichment method is the magnetic cell separation (MACS) (Miltenyi Biotec; Bergisch Gladbach, Germany). For CTC separation with the MACS kit, whole blood is first incubated with superparamagnetic beads coated with a target antibody, such as MCSP. The blood is then passed through a column containing ferromagnetic steel wool fibers, while a magnetic field captures the CTC-bead conjugates. Each column can process up to 15 mL of blood, and the CTCs are easily eluted once the magnetic field is removed (Miltenyi et al., 1990). Alongside its separation columns, Miltenyi has also developed the AutoMACS Pro Separator, a fully automated system that integrates in-device labeling with MACS reagents and magnetic separation (Zhu et al., 2018). Galanzha et al. used a MACS enrichment and detection kit with conjugated magnetic beads to isolate CTCs from the blood of melanoma patients, successfully detecting CTCs in those with relatively high concentrations (>1 CTC/mL) (Galanzha et al., 2019b).

#### 2.1.2 Negative enrichment

To minimize capture bias, some studies have used negative selection approaches, in which leukocytes are captured with antibody-coated beads and subsequently removed via magnetic separation. In most cases, anti-CD45 antibodies are conjugated to the magnetic beads to achieve leukocyte depletion (Xu et al., 2017). Two systems use the negative-enrichment technique. The first is EasySep (Stemcell Technologies, Vancouver, BC, Canada), which employs a magnetic field to capture leukocytes through a bispecific tetrameric antibody complex (TAC). This process leaves the supernatant containing a heterogeneous population of label-free CTCs (Liu et al., 2011). The second system is RosetteSep (Stemcell Technologies, Vancouver, BC, Canada). It uses a combination of antibodies targeting multiple blood cell markers (CD2, CD16, CD19, CD36, CD38, CD45, CD66b, and glycophorin A). When mixed with blood, these antibodies form a rosette network that traps unwanted blood cells. The rosetted cells are then removed via Ficoll density centrifugation, allowing the enrichment of the desired CTCs by depleting leukocytes and red blood cells (RBCs) (Fusi et al., 2011; He et al., 2008; Baccelli et al., 2013; Brungs et al., 2020; Rossi et al., 2025).

Fusi and colleagues used EasySep followed by melanoma CTC detection using gp100 and Melan-A (MLANA) via flow cytometry. They found CTCs in 28 out of 32 (87.5%) metastatic melanoma patients, with a median of 53 CTCs per 10 mL of blood (Fusi et al., 2011). Fankhauser et al. employed a variant of EasySep, called EasySep Direct (Stemcell Technologies, Vancouver, BC, Canada), which utilizes a TAC cocktail to label unwanted cells, platelets, and RBCs in whole blood, depleting them with magnetic particles in a single step. The magnetic antibodies cocktail (anti-CD2, CD14, CD16, CD19, CD45, CD61, CD66b, and Glycophorin A) enables simultaneous removal of erythrocytes and leukocytes without density gradient centrifugation or RBC lysis. The average recovery efficiency was 30.7% (Fankhauser et al., 2022).

Using RosetteSep, Girotti et al. successfully enriched CTCs and injected them into NOD scid gamma (NSG) mice to create cell line-derived xenograft (CDX) models. Although the exact number of CTCs was not determined, a substantial quantity of cells was likely isolated to ensure the tumor engraftment. Melanoma CDX models derived from these CTCs were used to study patient responses to trametinib, an inhibitor of the mitogen-activated protein kinases MEK1 and MEK2 (Girotti et al., 2016).

Another group used RosetteSep to separate erythrocytes and leukocytes from melanoma CTC combined with the S100-EPISPOT assay,and compared the relative results with those obtained with the CellSearch system. EPithelial ImmunoSPOT (EPISPOT) assay detects viable melanoma CTCs based on their secretion of the S100 protein (S100-EPISPOT). The combined approach demonstrated significantly higher sensitivity than the CellSearch system. Specifically, the percentage of patients with ≥2 CTCs was 48% with the S100-EPISPOT assay and 21% with the CellSearch system (*p value* = 0.0269) (Cayrefourcq et al., 2019).

#### 2.1.3 Microfluidic immunocapture

Microfluidic-based cell sorting methods involve the controlled flow of blood through a chip designed to capture CTCs, either by targeting cell surface markers (label-dependent immunocapture) or by cell size (label-independent, size-based enrichment). Immunocapture has been combined with microfluidics, flowing blood at a controlled rate over anti-EpCAM-coated walls or posts to increase cell contact and improve capture efficiency (Zou and Cui, 2018; Nasiri et al., 2020; Lin et al., 2021).

The CTC-Chip (Massachusetts General Hospital (MGH), US) is a silicon microfluidic platform, approximately the size of a standard microscope slide, featuring 78,000 anti-EpCAM-coated microposts. This design offers a large surface area for capturing CTCs. The CTC-Chip efficiently isolates viable CTCs from whole blood without sample pre-labeling or processing, which helps to maintain cell viability and activity and improve separation purity (Sequist et al., 2009; Nagrath et al., 2007). The CTC-Chip has been used in a Phase II study (NCT00888134) that investigated the use of Selumetinib in cancers with BRAF mutations, such as melanoma (Rushton et al., 2021).

CTC-Chip has also been enhanced into a new design featuring herringbone groove patterns, known as the Herringbone (HB) CTC-Chip. This design disrupts the blood flow within the channels, increasing capture efficiency by allowing more CTCs to come into contact with the antibodies lining the chip’s inner walls (Nagrath et al., 2007; Sharma et al., 2018; Sarioglu et al., 2015; Stott et al., 2010). The HB-CTC-Chip microfluidic platform has been adapted to capture melanoma CTCs using panels of antibodies targeting melanoma-specific cell surface markers, followed by immunofluorescence staining for melanoma antigens and optimized on-chip imaging. This platform was applied to a mouse melanoma model and a pilot cohort of human samples, leading to promising results. The integration of HB-CTC-chip technology with a panel of 12 melanoma-specific antibodies enabled the capture of CTCs (averaging 8 CTCs per 2.5 mL) through immunostaining in 32 out of 41 (79%) metastatic melanoma patients across different stages of treatment (Luo et al., 2014).

The Nanovelcro chip (University of California, Los Angeles (UCLA), US) uses an anti-EpCAM-coated silicon nanowire substrate (SiNS) to immobilize CTCs on the chip. Additionally, the chip features a polydimethylsiloxane (PDMS) chaotic mixer on the roof, which disrupts the laminar flow of blood and enhances contact between CTCs and EpCAM antibodies, improving capture efficiency (Jan et al., 2018). The second-generation nanovelcro-laser capture microdissection (LCM) chip is designed for single CTC isolation: it adds a CTC capture polymer substrate that can be dissected using an LCM microscope, allowing for the isolation of single CTCs for molecular analysis. The second-generation NanoVelcro Chip has been used to attempt single-CTC mutational analysis by detecting the BRAFV600E oncogenic mutation in individual melanoma CTCs. By employing melanoma-specific anti-CD146 as the capture agent, single CTCs have been isolated from several stage IV melanoma patients with known BRAFV600E mutations, confirmed by standard sequencing of tissue biopsies (Jan et al., 2018; Hou S. et al., 2013).

The IsoFlux system (Fluxion Biosciences, Inc., Alameda, CA, US) is a semi-automated platform that enriches CTCs through immunomagnetic positive selection and microfluidics. It offers significantly improved isolation efficiencies compared to the FDA-approved CellSearch (Cayrefourcq et al., 2019). Whole blood is introduced into the device, where it is incubated with antibody-coated magnetic beads. The blood then flows through a chip, and CTCs are captured in a cavity on the chip’s upper surface, which is exposed to an external magnetic field (Rushton et al., 2021; Agerbaek et al., 2018). IsoFlux CTC isolation platform, combined with magnetic beads targeting MCAM and MCSP, successfully isolated CTC from melanoma cultured cells and melanoma patient blood samples (Po et al., 2019).

#### 2.1.4 Nanomaterial-enhanced capture

To further enhance capture efficiency, various research groups have developed chips that also incorporate nanomaterials.

A multifunctional hybrid graphene oxide (GO) chip for label-free detection of malignant melanoma has been developed and used by Kanchanapally and colleagues since the tumor-associated disialoganglioside GD2 (GD2) is known to be uniformly expressed in most melanomas. An anti-GD2 antibody has been attached to the multifunctional graphene oxide chip for the separation of malignant melanoma cells (Kanchanapally et al., 2014; Pramani et al., 2018).

### 2.2 Biophysical property enrichment methods

Biophysical property enrichment methods used membrane filtration, size-based microfluidics, density-based techniques, and dielectrophoresis (Janjua et al., 2025; Rushton et al., 2021) (Table 2).

**TABLE 2:** List of biophysical property enrichment methods for CTC detection and isolation in melanoma.

Methods	Platform	Description	Advantages	Disadvantages	Ref.
Membrane filtration	Flexible Micro Spring Array (FMSA)	Filtration system made of round pores and flexible micro-spring structures	Very fast Isolation of viable CTCs High capture efficiency	Low sample purity Filter clogging	Harouaka et al. (2014)
	ScreenCell Cyto	Filter-based size-exclusion separation and enrichment of fixed or live CTCs	Rapid processing High recovery rate	Low sample purity Filter clogging	Desitter et al. (2011), Wechsler et al. (2012)
	Isolation by Size of Epithelial Tumor Cells (ISET)	Filter-based (size-/deformability exclusion separation) with 8 μm diameter cylindrical pores	Easy and rapid processing High sensitivity Isolation of EpCAM-negative CTCs Allows multiplexing	May miss cells < 8 μm in size Low recovery and purity Risk of false negatives due to the morphological variability of CTCs	Farace et al. (2011), Vona et al. (2000), De Giorgi et al. (2010a)
Size-based microfluidics	Parsortix Cell Separation System	Isolation of CTCs based on size and deformability	FDA approved* Isolation of viable CTCs	Low specificity and recovery rate	Miller et al. (2018), Aya-Bonilla et al. (2020),
	ClearCell FX1 System	Microfluidic separation based on centrifugal force and size of CTCs (≥14 μm diameter)	Isolation of viable CTCs Short processing time	Less efficient for small CTCs	Aya-Bonilla et al. (2020), Hou H. W. et al. (2013)
	VTX-1 Liquid Biopsy System	Isolation based on size, shape, and deformability using inertial microfluidics and laminar vortices	Automated isolation of viable CTCs High recovery rate	Low specificity and sample purity Risk of false negatives	Sollier-Christen et al. (2018), Martel et al. (2023)
Density-based methods	OncoQuick	Combined density-based gradient centrifugation and filtration	Rapid and cheap method Prevention of mixing cells Isolation of viable cells	Relatively low yield and enrichment	Rosenberg et al. (2002), Muller et al. (2005)
Dielectophoresis	DEP Microwell Array System	Capture of single CTCs through a high-density dielectrophoretic microwell array technology	High detection rate and high sensitivity	Expensive Potential cell damage	Morimoto et al. (2015), Kiniwa et al. (2021)

* for metastatic breast cancer.

#### 2.2.1 Membrane filtration

Membrane filtration-based methods for CTC capture offer advantages such as simplicity, speed, and high throughput (Dolfus et al., 2015; Han et al., 2022).

The flexible micro spring array (FMSA) consists of 8 μm round pores and flexible micro spring structures etched into a parylene filter. It is linked to a pressure regulation system that gently drives blood through the filter to preserve CTC viability, with reverse pressure applied to recover the cells. The FMSA device has been designed and used for the enrichment of viable CTCs independently of antigen expression. It has also been tested in melanoma and breast cancer cell lines (Harouaka et al., 2014).

The ScreenCell features filters with randomly distributed pores of 7.5 μm or 6.5 μm, designed for isolating fixed or live cells, respectively. The device requires an initial RBC lysis step but boasts nearly 100% cell recovery and a rapid processing time of just 50 s for 1 mL of blood (Desitter et al., 2011). ScreenCell has been used for the isolation of CTCs in patients with melanoma (Wechsler et al., 2012), and the clinical feasibility of CTC capture in patients with metastatic melanoma has been demonstrated (Yanagita et al., 2018). The ScreenCell device was utilized in the EXPEVIVO-CTC trial (NCT03797053) [102] for the *ex vivo* expansion of CTCs from melanoma patients, serving as a model for cancer predictive pharmacology (Rushton et al., 2021; Kaminska et al., 2021).

The Isolation by Size of Epithelial Tumor Cells (ISET) is a filtration-based device (Rarecells Diagnostics, Paris, France) and features 8 μm cylindrical pores, and can simultaneously process up to 12 samples. Unlike other membrane filters, it has a slower processing time. Blood is filtered using gentle vacuum aspiration. The device can process as little as 1 mL of blood, which is useful for those cancer patients for whom it is difficult to perform blood draws. It is also highly sensitive and capable of detecting a single tumor cell in 1 mL of blood. Additionally, the system includes a semi-automated CTC biopsy tool for easy enumeration after filtration (Rushton et al., 2021; Farace et al., 2011; Vona et al., 2000). Although ISET was developed to search for epithelial-derived cancer cells, it has also been used in uveal melanomas (Mazzini et al., 2014; Pinzani et al., 2010) and cutaneous melanoma patients (De Giorgi et al., 2010a), yielding promising results. De Giorgi et al. investigated the presence of CTC using ISET in the peripheral blood of 140 subjects, which included 87 melanoma patients (17 with *in situ* melanomas, 62 with primary invasive melanoma, and 8 with metastatic melanomas), 10 individuals undergoing surgery for benign nevi, 5 patients with non-melanoma skin tumors, and 38 healthy volunteers. The identification of cells trapped in filters as CTC was confirmed through positivity for immunohistochemical markers such as anti-S100 protein, MART-1, HMB-45, and tyrosinase mRNA via reverse transcriptase-PCR (RT-PCR). CTCs were found in 29% of primary invasive melanoma patients and 62.5% of metastatic melanoma patients, but were not detected in the controls or in the *in situ* melanoma group. Additionally, CTC detection was correlated with the presence of tyrosinase mRNA in blood samples (De Giorgi et al., 2010a). However, a case report showed that this approach has also identified benign circulating nevus cells (De Giorgi et al., 2010b), indicating that the assay is unable to differentiate between benign nevus cells and melanoma cells. Khoja et al. used ISET to isolate CTC, which were quantified using dual immunohistochemistry with S100 expression for positive selection, while excluding leukocytes and endothelial cells marked with CD45 and CD144 antigens, respectively. Additional melanoma markers, such as Melan-A, MITF, MelCAM, CD271, MAGEC, were also evaluated. Of the 51 patients (57%) with detectable CTCs, the count ranged from 1 to 44 CTCs per 4 mL of blood, and 12 patients had circulating tumor microemboli. The presence of both S100+ and S100- CTCs varied across patients, revealing significant marker expression heterogeneity and suggesting the limitations of marker-dependent platforms and the need for multimarker assays to better understand CTC heterogeneity (Khoja et al., 2014).

#### 2.2.2 Size-based microfluidics

Size-based microfluidic devices are promising for the next-generation of CTC enrichment technologies, as they separate CTCs without relying on cell surface markers, potentially capturing the full range of CTCs across various cancers. These devices exploit the fact that CTCs (8–30 μm) are typically larger and less deformable than leukocytes (12–15 μm). However, due to the overlap in size between CTCs and leukocytes, optimizing these devices is essential to maximize CTC recovery while minimizing leukocyte contamination (Rushton et al., 2021).

The Parsortix Cell Separation System (Angle plc, Surrey, United Kingdom) device is a semi-automated platform designed to capture and harvest rare cells, such as CTCs, from bodily fluids like blood, bone marrow, and ascites, based on their size and deformability (or compressibility) and is the first FDA-cleared medical device for the capture and harvest of CTCs from peripheral blood of metastatic breast cancer (U.S. FOOD & DRUG ADMINISTRATION, 2022). The system operates without the use of antibodies or other cell surface affinity agents to isolate the target cells with a recovery rate of 79%–89% for melanoma CTCs (Miller et al., 2018; Aya-Bonilla et al., 2020). Parsortix has also been used to enrich disseminated cancer cells from sentinel lymph nodes. The optimized workflow achieves a recovery rate of over 60% of DCCs from melanoma patient-derived lymph node suspensions. The advantage is that Parsortix allowed the isolation of viable DCCs for further molecular analysis (Weidele et al., 2019).

Poggiana and colleagues evaluated CellSearch and Parsortix platforms and investigated their combined application for CTC enumeration in melanoma. Using a custom melanoma antibody cocktail, Parsortix effectively distinguished CTCs from blood cells. Although both platforms showed comparable capture rates for cell lines, Parsortix outperformed CellSearch in patient-derived samples. Furthermore, sequential enrichment with both systems substantially reduced leukocyte contamination, producing nearly pure CTC populations (Poggiana et al., 2025).

Parsortix has been compared in 43 blood samples from patients with metastatic melanoma with another microfluidic device for the recovery of circulating melanoma cells (Aya-Bonilla et al., 2020), namely the ClearCell FX1 platform (Biolidics, Singapore), which is a spiral microchannel with inherent centrifugal forces for continuous, size-based separation of CTCs from blood (Hou H. W. et al., 2013). The collected CTCs were evaluated using a combination of immunocytochemistry and transcript analyses of five genes by RT-PCR (MLANA, TYR, MAGEA3, ABCB5, PAX3) and 19 genes by droplet digital PCR (ddPCR) (MCSP, FAT1, FAT2, GAGE1, GPR143, IL13RA2, MAGEA1, MAGEA2, MAGEA4, MAGEA6, MAGEC2, MLANA, PMEL, PRAME, SFRP1, SOX10, TFAP2, TNC, TYRP1). ClearCell demonstrated a slightly better performance compared to Parsortix, by detecting melanoma-specific transcripts in two additional CTC samples (Aya-Bonilla et al., 2020). The ClearCell FX technology is optimized to enrich cells with a diameter of approximately 14 μm or larger. This parameter is effective for enriching CTCs from tumor types like breast cancer and melanoma, which tend to have relatively larger CTCs. However, it may be less efficient for tumors with smaller cell types, such as colon cancer and small-cell lung cancer (Lee et al., 2018).

VTX-1 (Vortex Biosciences, CA, US) technology utilizes inertial microfluidics, employing laminar microscale vortices to isolate and concentrate CTCs from blood. CTC capture is based on cell size, shape, and deformability, and does not rely on tumor biomarkers. The VTX-1 Liquid Biopsy System automates the isolation of clinically significant CTC populations, streamlining their collection for easier analysis and broadening the clinical applications of CTCs (Sollier-Christen et al., 2018). A study compared the efficiency of VTX-1 with other three platforms, namely, ClearCell FX, ISET, and CellSearch, in detecting and identifying uveal melanoma cells. The mean overall recovery rates (along with the mean number of recovered cells) were 39.2%, 22.2%, 8.9%, and 1.1% for ISET, VTX-1, ClearCell FX, and CellSearch platforms, respectively (Martel et al., 2023).

#### 2.2.3 Density-based

Density-based separation methods represent a simpler category of CTC enrichment technologies. As one of the earliest techniques developed for CTC separation, they have become less favored over time, as advancements in technology have made other CTC separation methods more efficient (Rushton et al., 2021).

The OncoQuick (Greiner Bio-One, Austria) system is a size-based technique that combines filtration with density-based centrifugation for CTC separation (Rosenberg et al., 2002; Maltoni et al., 2015). The centrifugation tube features a micro-filter positioned above the liquid density separation medium. Blood is layered on top of the gradient, and during centrifugation, CTCs are captured on the filter (Muller et al., 2005). Clawson et al. collected blood samples from melanoma patients at different disease stages and from healthy controls. After fractionation with OncoQuick columns, RNA was extracted from the resulting CTC-enriched fractions. qPCR analysis after OncoQuick enrichment revealed that approximately one-third of early-stage patients had higher levels of macrophage migration inhibitory factor (MIF) and MLANA transcripts compared to healthy controls (*p value* < 0.0001 and *p value* < 0.001, respectively) (Clawson et al., 2012).

#### 2.2.4 Dielectrophoresis

Enrichment techniques can employ dielectrophoretic field forces to selectively move CTCs apart from other blood cells. This method does not require labeling, enables separation regardless of EpCAM expression, and is highly specific, although it tends to be expensive. Morimoto et al. developed a high-density dielectrophoretic microwell array for the detection and capture of rare tumor cells in peripheral blood (Morimoto et al., 2015).

Kiniwa et al. applied this method to melanoma blood samples, isolating CTCs from peripheral blood using a high-density dielectrophoretic microwell array. The cells were then labeled with melanoma-specific markers, namely MART-1 and/or gp100, and CD45. MART-1/gp100-positive and CD45-negative cells counted as CTCs. CTC counts were analyzed in fifteen stage 0–III melanoma patients. Additionally, changes in CTC numbers were assessed at four time points during BRAF/MEK inhibitor treatment in five stage IV melanoma patients. CTCs were present even in the early stage of melanoma, and in four out of five stage IV melanoma patients, CTC numbers varied with BRAF/MEK inhibitor treatment, indicating that CTC count could serve as a potential biomarker for drug response in advanced disease (Kiniwa et al., 2021).

### 2.3 Combined methods

To further enhance the isolation of rare CTC populations, new microfluidic strategies have been developed that combine physical and immunological approaches (Table 3).

**TABLE 3:** List of combined methods for CTC detection and isolation in melanoma.

Platform	Description	Advantages	Disadvantages	Ref.
CTC-iChip	A microfluidic device that isolates viable, label-free circulating tumor cells by combining size-based sorting with immunomagnetic depletion of white blood cells	Allows the sequential separation of different blood components immunomagnetically labeled Isolation and characterization of CTCs with both epithelial and mesenchymal traits	Low sample purity Expensive Long set-up time	Janjua et al. (2025), Ozkumur et al. (2013)
Spiral microfluidic device	A microfluidic device based on CTC size and physical properties	Isolation of viable CTCs High recovery efficiency	Possible white blood cell contamination Risk of clogging	Khoo et al. (2014), Aya-Bonilla et al. (2017)

The CTC-iChip, developed by MGH as a third-generation chip (following the CTC-chip and HB-chip), employs a combination of multiple methods for CTC enrichment (Karabacak et al., 2014). Specifically, it combines tumor antigen-dependent and independent methods, integrating hydrodynamic size-based separation with automated monolithic chips to achieve high capture efficiency and cell viability, virtually enabling the isolation of CTC from all cancers (Janjua et al., 2025). The CTC iChip offers efficient white blood cell (WBC) depletion and enables the characterization of CTCs with both epithelial and mesenchymal traits (Zhang et al., 2018). It combines size-based enrichment with either EpCAM-based positive selection or CD45 negative depletion, achieving 97% capture yield and processing rates of 8 mL/h. It removes unnucleated cells using a micropost array and eliminates WBC through antibody-mediated methods, resulting in label-free, viable CTCs. However, EpCAM-based positive selection is limited by the heterogeneous and often downregulated expression of EpCAM on CTCs and it can only isolate single CTCs and small clusters (2-4 cells) (Sharma et al., 2018; Ozkumur et al., 2013; Karabacak et al., 2014). Using CTC-iChip, CTCs from two metastatic melanoma patients were successfully enriched and detected by staining for MLANA (Ozkumur et al., 2013).

Some variants of the CTC-iChip have also been developed. The monolithic CTC-iChip is an automated, fully integrated plastic chip that isolates CTCs regardless of size or epitope. However, neither CTC size nor EpCAM expression alone can optimize isolation efficiency, as many CTCs are small and express lower levels of EpCAM (Fachin et al., 2017). The Ephesia CTC-Chip integrates immunobead technology with microfluidics, enabling rapid isolation and enumeration of CTCs with 90%–94% capture efficiency and non-specific capture rates under 0.4%. A key challenge remains to enhance its processing capacity for larger sample volumes (Karabacak et al., 2014; Alix-Panabieres et al., 2012; Saliba et al., 2010).

It is worth noting that the CTC-iChip, when used in EpCAM-based positive selection mode, may underperform in melanoma due to the non-epithelial origin of melanocytes and the frequent lack of EpCAM expression on melanoma CTCs. In this context, CD45-negative depletion mode is generally preferable, as it enables capture of EpCAM-negative CTCs and better reflects the phenotypic diversity of circulating melanoma cells.

Spiral microfluidic devices have been recently developed to isolate CTCs based on cell size, deformability, and density, achieving high purity, viability, and recovery efficiency (Omrani et al., 2023; Herrmann et al., 2019). These devices have outperformed the FDA-approved CellSearch platform in recovering CTCs from breast and lung cancer patients. They can process 8 mL of blood in less than 10 min, yielding CTCs with relatively high purity (500 WBCs per mL), representing a significant improvement over other devices that take over an hour (Khoo et al., 2014). The use of the slanted spiral microfluidic device has been validated to isolate viable, label-free, and heterogeneous CTC populations from the blood of metastatic melanoma patients. They showed that the device achieved recovery rates above 80% after one enrichment round and around 55% after two rounds for spiked melanoma cells, while simultaneously reducing white blood cells by two to three orders of magnitude (Aya-Bonilla et al., 2017).

### 2.4 *In Vivo* enrichment methods

To supplement conventional CTC isolation platforms, *in vivo* technologies have been used to increase detection sensitivity and expand opportunities for detailed characterization (Table 4).

**TABLE 4:** List of *In Vivo* enrichment methods for CTC detection and isolation in melanoma.

Methods	Description	Advantages	Disadvantages	Ref.
Diagnostic leukapheresis (DLA)	Isolation of CTCs within the PBMCs fraction from large volumes of blood	Increased CTC detection Large blood volumes	Invasiveness limits routine use	Fischer et al. (2013), Bos et al. (2024)
Photoacoustic flow cytometry platform (PAFC)	*In vivo* detection of acoustic waves from melanin-bearing CTCs by transcutaneous delivery of laser pulses to blood vessels	High sensitivity Identification of CTC-clusters Analysis of large volumes of blood Noninvasive	High false-negative rate Time-Consuming Optical Clearing Need for External Contrast Agents for Non-Melanoma Tumors	Galanzha et al. (2019a) Galanzha et al. (2019b)

Diagnostic leukapheresis (DLA) is a technique that screens large volumes of whole blood and can serve as a pre-enrichment step to address the low CTC numbers typically found in small blood samples; in fact, leukapheresis targets peripheral blood mononuclear cells, which have a similar density to CTCs. DLA permits sensitive detection of rare CTCs from large blood volumes, enabling detailed biological and functional insights, while its invasiveness limits routine use, making it most valuable for guiding personalized therapies and high-volume liquid biopsy applications (Fischer et al., 2013).

A study explored whether combining DLA with FCM improved CTC detection in melanoma patient blood compared to the CellSearch platform. The results showed that DLA generally increased the proportion of patients with detectable CTCs, but it was also linked to a low recovery rate (Bos et al., 2024).

In the study of 20 patients with metastatic melanoma, Bos et al. used DLA combined with multimarker flow cytometry (FCM), which contained antibodies against CD45, MCSP, CD146, and the nuclear dye DRAQ5 as a backbone to identify melanoma CTCs, and increased the proportion of patients with detectable CTCs from 35% (CellSearch on peripheral blood) to 70% (*p value* = 0.06). However, the median recovery rate of cells through the DLA procedure was 29% (Bos et al., 2024).

Cytophone technology identifies rare biomarkers by examining large volumes of blood *in vivo* using the photoacoustic flow cytometry platform (PAFC) with a high-pulse-rate laser and focused ultrasound transducers for label-free detection of melanin-bearing CTCs in melanoma patients. Laser pulses are delivered transcutaneously to a blood vessel, generating acoustic waves from CTCs, which are enhanced by vapor nanobubbles around melanin nanoclusters. Time-resolved acoustic wave detection, supported by fast signal processing, ensures tolerance to skin pigmentation and motion. No CTC signals were detected in 19 healthy volunteers, but 27 of 28 melanoma patients showed signals consistent with single, clustered, and possibly rolling CTCs. PAFC shows strong sensitivity and can operate label-free for naturally pigmented targets, with promising applications in detecting circulating blood cells for various diseases. However, its use is limited by false negatives in low-melanin CTCs, and the need for contrast agents for non-melanoma cancer (Galanzha et al., 2019a; Galanzha et al., 2019b).

### 2.5 Secondary isolation technologies

The isolation technologies and platforms described above often leave residual leukocyte contamination, which can affect and alter molecular analysis of CTCs. To enhance the specificity of molecular characterization, an additional isolation step may be necessary to remove contaminating WBCs, whose DNA or RNA can interfere with analysis. This secondary step allowed the isolation of single CTCs for studying intra-patient heterogeneity or obtaining pure CTC populations. Primary isolation methods should be combined with single-cell sorting technologies, such as the CellCelector, and the DEPArray, which are already used in tumor research alongside primary enrichment techniques (Rushton et al., 2021; Ortiz and Yu, 2018). Both technologies provide versatile and efficient solutions for CTC isolation and downstream analysis.

The CellCelector (Automated Lab Solutions; Jena, Germany) is an automated platform that uses micromanipulation with a high-precision glass micro-capillary attached to a robotic arm to isolate individual CTCs from whole blood samples (Nelep and Eberhardt, 2018), working with various primary enrichment methods, such as Cell Search (Kostler et al., 2024; Lampignano et al., 2017; Neumann et al., 2017), MagSweeper (Lohr et al., 2014), RosetteSep (Yao et al., 2014) and Parsortix (Lampignano et al., 2017).

The DEPArray (Menarini Silicon Biosystems), a microchip-based digital sorter, uses microfluidics and dielectrophoresis to isolate single CTCs, offering a high-precision tool with the ability to visualize cells through multiple fluorescent channels (Fontana et al., 2017; Di Trapani et al., 2018). The DEPArray is part of a standardized and reproducible workflow that includes CellSearch primary enrichment, DEPArray single-cell sorting, and Ampli1 whole genome amplification (WGA) analysis for precise molecular characterization of heterogeneous single CTCs (Polzer et al., 2014).

CTCs from 33 metastatic melanoma patients were isolated with the CellSearch/DEPArray protocol, and mutations were compared with those from the tumor tissue and circulating tumor DNA (ctDNA). While ctDNA showed better concordance with tumor tissue, CTCs contained a larger number of mutations, including those in melanoma driver genes and genes associated with therapy resistance or metastasis (*p value* = 0.039). These results demonstrate that CTC analysis can provide clinically relevant genomic data that complements, rather than duplicates, tumor tissue or ctDNA analysis (Sementsov et al., 2024).

The DEPArray system was used to isolate CTCs from 17 stage IV melanoma patients following immune-magnetic depletion of blood (CD45) and endothelial cells (CD31, CD34). Analysis of stem cell (CD271, ABCB5, Receptor activator of nuclear factor κ B (RANK)) and mesenchymal (N-cad, CD44, MCAM/CD146) markers showed that most CTCs exhibited mesenchymal traits alone or combined with stemness, consistent with invasiveness. The analysis revealed significant immune phenotype heterogeneity, correlating with clinical outcomes (Tucci et al., 2020).

### 2.6 Molecular CTC detection

Although the aforementioned platforms have improved CTC isolation, molecular techniques such as immunocytochemistry (ICC), flow cytometry, and gene expression analyses remain essential for the accurate identification and characterization of CTCs.

The complementary roles of ICC, flow cytometry, and gene expression analyses in detecting and characterizing melanoma CTCs highlight the heterogeneity of these cells and the potential to use molecular markers for prognostic and therapeutic purposes.

#### 2.6.1 Immunocytochemistry and flow cytometry

ICC and flow cytometry can be applied to identify CTCs based on their morphology and protein expression.

ICC allows the detection of a single tumor cell among 10,000 to 100,000 non-tumor cells by combining fluorescently labeled monoclonal antibodies against specific tumor antigens with automated imaging systems. Despite its sensitivity, ICC is limited by the number of cells that can be analyzed, potential cross-reactivity, and lower sensitivity compared to molecular assays such as RT-PCR (Vidlarova et al., 2023). Flow cytometry provides an objective method for analyzing suspended particles, enabling differentiation and characterization of cells based on surface antigens. Antibody labeling allows precise identification of phenotypic markers on target populations, and because CTCs express specific surface markers, flow cytometry can detect them in body fluids, often before visualization by imaging (Gostomczyk et al., 2024).

Ruiz and colleagues analyzed blood samples from 40 metastatic melanoma patients using a panel of seven monoclonal antibodies targeting chondroitin sulfate proteoglycan 4 (CSPG4) for immunocytochemical CTC detection. This approach identified 1–250 CTCs per 8 mL of blood in 55% of patients and allowed whole-genome analysis, revealing gene deletions and amplifications associated with melanoma progression (Ruiz et al., 2015).

A flow cytometry-based multimarker approach has been developed to detect melanoma-associated markers, namely MCSP, MCAM, and melanoma stem cell markers, namely ABCB5, RANK, and CD271, in CTCs. This method showed that a high proportion of CTCs expressed stem cell–related markers (ABCB5, RANK), while only a small fraction expressed MSCP and MCAM. Notably, the expression of these markers on CTCs did not correlate with their expression in matched tumors, suggesting that most melanoma CTCs originate from rare subpopulations with metastatic potential, rather than from bulk tumor cells (Gray et al., 2015).

#### 2.6.2 Gene expression analysis

CTCs exhibit distinct gene expression profiles from white blood cells, enabling their detection in blood samples via RT-qPCR (Andergassen et al., 2016; Guo et al., 2015) and ddPCR (Denis et al., 2016).

CTCs in melanoma were initially studied by detecting tyrosinase, which is normally absent in peripheral blood, using RT-PCR (Smith et al., 1991; Brownbridge et al., 2001; Curry et al., 1998; Hoon et al., 1995).

Multiple-marker RT-PCR assays targeting melanoma-specific transcripts has since become a sensitive and reliable method for identifying CTCs in blood and lymph nodes (Curry et al., 1998; Rapanotti et al., 2017; Frank et al., 2003). The most frequently investigated mRNA markers include MLANA (Brownbridge et al., 2001; Curry et al., 1998; Hoon et al., 1995; Schittek et al., 1999), beta-1,4-N acetyl-galactosaminyl transferase 1 (B4GALNT1), melanoma-antigen -family A3 (MAGE-3) (Curry et al., 1998; Hoon et al., 1995; Gaugler et al., 1994), melanotransferrin (MFI2, p97) (Curry et al., 1998; Hoon et al., 1995), MCAM (Curry et al., 1998; Hoon et al., 1995; Lehmann et al., 1987; Melnikova and Bar-Eli, 2006), tyrosinase-related protein 1 (TRP-1) and 2 (TRP-2) (Jimenez-Cervantes et al., 1994; Sarantou et al., 1997), MCSP (Yang et al., 2009), ABCB5 (Frank et al., 2003; Schatton and Frank, 2008; Vendittelli et al., 2015).

Using immunomagnetic beads against MCSP, ABCB5, MAGEA3, RANK, or a combination of these cell surface antigens, CTCs from blood samples of metastatic melanoma patients with confirmed BRAF mutation-positive tumors were isolated, and subsequently RNA and DNA were extracted from CTCs. RT-PCR of RNA was employed to confirm the presence of melanoma cells in the CTC fraction. DNA from the CTC-positive fractions was then subjected to WGA and tested for BRAF V600E or V600K mutations via ddPCR (Reid et al., 2015).

A promising telomerase-based assay for CTC detection was developed, leveraging the elevated telomerase activity commonly observed in melanoma cells. This assay uses an adenoviral vector that, in the presence of increased human telomerase activity, drives the amplification of green fluorescent protein. Tumor cells were subsequently identified using an image processing system. Genetic analysis of isolated melanoma CTCs from metastatic melanoma patients was performed to assess BRAF mutation status. CTCs were detected in 9 out of 10 patients, with a mean of 6.0 CTCs/mL. Using a cutoff of 1.1 CTCs/mL, the telomerase-based assay demonstrated 90.0% sensitivity and 91.7% specificity. BRAF mutation analysis of pilot patient samples was consistent with the known BRAF mutation status of their primary tumors (Xu et al., 2015).

#### 2.6.3 Combined approaches

Aya-Bonilla et al. demonstrated that a slanted spiral microfluidic device can isolate viable, label-free CTCs from metastatic melanoma patients, enriching heterogeneous subpopulations. Secondly, melanoma CTCs were characterized using flow cytometry, gene expression analysis, and immunostaining. Flow cytometry revealed preferential recovery of ABCB5+ cells, either alone or in combination with RANK, a marker associated with treatment resistance; while immunostaining detected melanocytic markers (gp100, S100, and MLANA) in 43% of patients. Gene expression analysis confirmed melanoma-specific transcripts, including Paired Box 3 (PAX3), ABCB5, and MLANA, in 54% of CTC fractions (Aya-Bonilla et al., 2017), with PAX3 and ABCB5 associated with metastatic disease and potential therapy resistance (Gostomczyk et al., 2024).

## 3 Clinical role of CTCs in melanoma

CTCs were first described in 1869 by Thomas Ashworth in a patient with metastatic cancer (Ashworth, 1869; Galvis et al., 2021). In melanoma, molecular detection of CTCs was first reported in 1991, when Smith et al. identified melanoma cells in peripheral blood using a tyrosinase-specific RT-PCR assay (Smith et al., 1991). Since then, numerous studies have investigated CTC detection in melanoma across different disease stages. CTCs can be detectable at every stage of melanoma, including melanoma *in situ,* and can remain present long after treatment. Therefore, the detection of CTCs could help monitor disease progression and predict patient outcomes (Kiniwa et al., 2021; Rapanotti et al., 2017; Mumford and Robertson, 2014).

### 3.1 CTCs as a diagnostic marker

CTCs from melanoma patients across different disease stages were detected even in early-stage melanoma (stages 0–I), and a high CTC count in one stage I patient preceded lymph node recurrence 2 years later (Kiniwa et al., 2021). Fourdrain et al. enrolled 35 melanoma patients (9% stage II, 54% stage III, and 37% stage IV) and showed that all patients had detectable CTCs at baseline, supporting the concept that tumor cells circulate in the bloodstream across all stages of melanoma. Although CTCs were detected at every stage, their abundance varied significantly according to AJCC stage, with markedly higher counts in metastatic than in non-metastatic disease (Fourdrain et al., 2025).

De Giorgi et al. detected CTCs in 63% of patients with metastatic melanoma and in 29% of those with primary invasive melanoma, while no CTCs were found in controls or patients with *in situ* melanoma (De Giorgi et al., 2010a).

Similarly, Freeman et al. reported the presence of CTCs across all disease stages, indicating early tumor cell dissemination (Freeman et al., 2012). However, significantly higher counts were observed in stages III–IV patients compared to those in stages I–II, highlighting the potential of CTC quantification for monitoring disease progression and identifying patients at risk (Freeman et al., 2012). Roland et al. found at least one CTC in 45% of melanoma patients, with detection rates increasing from 29% in stage I to 86% in stage IV (Roland et al., 2015). In the study by Hida et al., CTC detection alone had a 33% efficacy in identifying metastases in patients with stages II-IV melanoma, which increased to 67% when combined with additional biomarkers such as 5-S-cysteinyldopa (Hida et al., 2016).

These findings suggest that CTCs can emerge early in melanoma progression and may assist in diagnosis even at initial stages. Nevertheless, their clinical utility as a diagnostic tool remains inconsistent. This variability is primarily attributed to the cellular heterogeneity of CTCs and the wide range of detection platforms employed across studies, which vary in sensitivity and specificity. Consequently, these discrepancies can lead to both false positives and false negatives, limiting the diagnostic reliability of CTCs in clinical practice (Kaminska et al., 2021).

### 3.2 CTCs as a prognostic marker

In recent years, CTCs have emerged as a promising biomarker for the detection of metastasis, the monitoring of treatment response, and the prediction of prognosis in melanoma (Hida et al., 2016). CTC counts in peripheral blood are associated with clinical outcomes such as overall survival (OS), progression-free survival (PFS), or recurrence-free survival (RFS) or disease-free survival (DFS) (Nguyen et al., 2023). Several studies demonstrated that a higher total count of CTCs is associated with treatment failure and worse prognosis (Khoja et al., 2013). Conversely, baseline low CTC count or a decrease in CTC count during treatment was associated with a prolonged PFS (Klinac et al., 2014).

In the study of Bos et al., the presence of CTCs in leukapheresis samples was significantly associated with overall tumor burden (*p value* < 0.001), while CTCs detected in peripheral blood correlated with the number of lesions observed on radiological imaging (*p value* < 0.001) (Bos et al., 2024). Gray et al. developed a flow-cytometry-based approach to detect and analyze CTCs using both melanoma-associated markers (MCAM and MCSP and stemness markers (ABCB5, CD271, RANK), evidencing that the prognostic utility of melanoma CTC analysis depends not only on the total number but also on the presence of specific subpopulations. CTCs resulted in heterogeneous within and between patients, with limited co-expression between the five markers analyzed, and ABCB5+ and RANK+ subpopulations were found to be more prevalent with RANK+ CTCs particularly predictive of relapse following adjuvant BRAF inhibitor therapy (Gray et al., 2015). Aya-Bonilla and colleagues evaluated CTCs in metastatic melanoma patients using a combination of immunocytochemistry and transcript analysis via ddPCR to generate a molecular CTC score. They found that patients with a high CTC score had significantly shorter OS and PFS (Aya-Bonilla et al., 2020). Moreover, CTC presence has been correlated with melanoma relapse. Lucci et al. assessed CTCs in 243 patients with stage III melanoma using the CellSearch system, showing that one or more CTCs at baseline was significantly associated with a shorter RFS (Lucci et al., 2020). Similarly, in a pilot study of 93 stage IV melanoma patients, Hall et al. observed CTCs in 39/93 patients, and their presence was associated with disease progression within 180 days (Hall et al., 2018).

Overall, these findings highlight the prognostic relevance of CTC enumeration in melanoma, supporting the potential role in patient stratification.

### 3.3 CTCs as a monitoring marker

CTC count is associated with primary tumor progression, making longitudinal monitoring of CTC levels a valuable tool for evaluating therapeutic response. In therapeutic drug monitoring, CTCs offer a real-time, non-invasive biomarker for evaluating treatment effectiveness. Typically, a reduction in CTC levels after surgery, chemotherapy, or radiotherapy reflects a favorable response, such as tumor regression or disease control. On the other hand, an increase of CTCs potentially indicates treatment failure or tumor progression and the need for therapy adjustments (Nguyen et al., 2023).

In metastatic melanoma, Aya-Bonilla et al. demonstrated that CTC scores correlate with ctDNA concentrations at the start of therapy. In addition, they analyzed the dynamic changes of CTC molecular signatures upon treatment initiation and demonstrated that CTCs could provide information on tumor progression (Aya-Bonilla et al., 2020). Similarly, Hong et al. developed a 19-gene digital RNA signature (CTC score) from enriched melanoma CTCs to quantify early tumor response, demonstrating that a decrease in the CTC score within 7 weeks of immunotherapy was significantly associated with improved outcomes (Hong et al., 2018). In a cohort of 17 stage IV melanoma patients, Scaini et al. applied a combined approach of molecular assays to both ctDNA and CTCs. Although the absolute CTC count alone did not serve as a predictive biomarker of treatment response, molecular profiling of CTCs proved to be useful in the management and real-time monitoring of melanoma patients (Scaini et al., 2024). Khoja et al. assessed the prevalence of CTCs in 101 patients with metastatic melanoma using the CellSearch platform at baseline and during treatment. They identified a threshold of 2 CTCs per 7.5 mL of blood as an independent prognostic factor for OS. Moreover, longitudinal analysis in 45 treated patients revealed that all individuals exhibiting a decrease in CTC count experienced radiological responses, whereas patients whose CTC count remained ≥2 during treatment had a shorter median OS compared to those maintaining <2 CTCs (Khoja et al., 2013).

Collectively, these observations reinforce the concept that dynamic changes in CTC levels could be associated with treatment response and disease progression in melanoma.

## 4 Advances, challenges, and future perspectives

Despite major therapeutic improvements in melanoma, several unmet clinical needs persist, including improved risk stratification within the same disease stage, real-time assessment of treatment response, and early identification of disease progression or therapeutic resistance (Tarhini and Kudchadkar, 2018; Pilla et al., 2020; Hassel et al., 2023; Roccuzzo et al., 2024).

A critical and underappreciated limitation specific to melanoma is the non-epithelial origin of melanocytes, which renders EpCAM-based capture strategies inherently inadequate for this tumor type. Unlike carcinomas, melanoma CTCs frequently lack EpCAM expression, and reliance on EpCAM-dependent platforms risks systematic underestimation of circulating melanoma cells, particularly in early-stage disease where CTC abundance is already low.

Although technological advances have been made, clinical translation of CTCs in melanoma remains limited by methodological heterogeneity and lack of standardization (Marsavela et al., 2018; Guo and Xia, 2024). Current platforms employed to study CTCs in melanoma patients, including immunoaffinity-based CellSearch kits targeting MCAM/MCSP (Marsavela et al., 2018; Ligthart et al., 2011; Rao et al., 2011), MACS (Miltenyi et al., 1990; Galanzha et al., 2019b), EasySep (Liu et al., 2011; Fusi et al., 2011), RosetteSep (Girotti et al., 2016; He et al., 2008), biophysical methods such as FMSA, ScreenCell, ISET (Rushton et al., 2021; Harouaka et al., 2014; Wechsler et al., 2012; De Giorgi et al., 2010a; Yanagita et al., 2018; Kaminska et al., 2021), size-based microfluidic platforms (Parsortix, ClearCell FX1, VTX-1) (Miller et al., 2018; Aya-Bonilla et al., 2020; Hou H. W. et al., 2013; Martel et al., 2023; Weidele et al., 2019; Poggiana et al., 2025; Lee et al., 2018), and density-gradient separation platform (OncoQuick) (Clawson et al., 2012), capture complementary but non-overlapping CTC subsets, and most cannot simultaneously achieve both high recovery and high purity. No single platform captures the full phenotypic spectrum of melanoma CTCs, and the low abundance of CTCs in peripheral blood further limits detection sensitivity, resulting in significant variability in reported detection rates across studies. These limitations severely restrict their integration into large-scale clinical trials and, at present, preclude their use as a standardized clinical tool (Guo and Xia, 2024). Establishing consensus procedures for CTC enrichment, detection, and quantification across laboratories is a prerequisite for any future clinical implementation (Marsavela et al., 2018).

CTCs are unlikely to become a reliable standalone diagnostic tool in melanoma. Although CTCs can be detected across all disease stages, including early-stage melanoma and even melanoma *in situ* (Freeman et al., 2012; Kiniwa et al., 2021; Roland et al., 2015), and may precede clinical relapse (Kiniwa et al., 2021), their diagnostic reliability remains limited. In fact, detection rates vary widely across studies and disease stages (De Giorgi et al., 2010a; Fourdrain et al., 2025), largely due to CTC heterogeneity and the use of heterogeneous detection platforms with differing sensitivity and specificity (Kaminska et al., 2021).

In contrast, the prognostic value of CTCs is consistently supported across independent cohorts and methodologies. Multiple studies consistently report associations between higher baseline CTC counts and treatment failure, and worse survival outcomes, including OS, PFS, RFS, and DFS (Khoja et al., 2013; Lucci et al., 2020; Hall et al., 2018; Aya-Bonilla et al., 2020; Bos et al., 2024; Nguyen et al., 2023; Klinac et al., 2014). Interestingly, emerging evidence indicates that a specific CTC subpopulation (RANK+ CTCs), rather than total CTC count alone, may carry stronger prognostic significance (Gray et al., 2015).

Despite consistent associations between CTC counts and survival outcomes across independent cohorts, the lack of prospective validation studies and standardized enumeration thresholds currently prevents the incorporation of CTCs into clinical staging or treatment decision algorithms.

Longitudinal monitoring of CTCs represents a promising clinical application. Decreases in CTC counts following treatment are associated with favorable outcomes, whereas persistently elevated or increasing CTC levels predict disease progression and shorter survival (Khoja et al., 2013; Nguyen et al., 2023). Importantly, studies incorporating molecular profiling of CTCs show that dynamic changes in CTC molecular signatures correlate with treatment response and clinical outcomes (Aya-Bonilla et al., 2020; Hong et al., 2018; Scaini et al., 2024), suggesting that the biological features of CTCs may be more clinically informative than absolute counts alone.

Given the heterogeneity of available CTC detection approaches, Table 5 provides a comparative overview of their technical characteristics and translational potential, including feasibility, reproducibility, clinical applicability, and stage of development.

**TABLE 5:** Clinical applicability, reproducibility, and current limitations of CTC platforms in cutaneous melanoma.

Platform	Reproducibility in cutaneous melanoma	Cost and feasibility	Main barrier to clinical use in melanoma	Clinical implementation stage
CellSearch (MCAM/MCSP kit)	Moderate to high: standardized automated platform; limited melanoma-specific reproducibility data	Moderate; dedicated instrument required; compatible with standard laboratory infrastructure	Melanoma-specific MCAM/MCSP kit not FDA-cleared for melanoma; prognostic thresholds not yet validated across independent multicenter cohorts	Closest to clinical implementation
ISET	Moderate; no inter-laboratory study in cutaneous melanoma	Low; no dedicated instrument required	Risk of confounding with benign circulating melanocytic cells in patients with atypical nevi; no validated enumeration threshold in cutaneous melanoma	Clinically explored
ScreenCell	Moderate; no inter-laboratory standardization in cutaneous melanoma	Low; no dedicated instrument required	Limited validation and lack of standardized enumeration thresholds in cutaneous melanoma	Clinically explored
Parsortix	Moderate; analytical performance characterized in multiple solid tumors, but no melanoma-specific inter-laboratory standardization study	Moderate to high; dedicated instrument and single-use cassettes required	No melanoma-specific regulatory clearance or prospective multicenter validation	Clinically explored
ClearCell FX1	Not established in cutaneous melanoma	Moderate to high; dedicated instrument required	No regulatory clearance; no independent replication in cutaneous melanoma	Research stage
Slanted spiral microfluidic	Not established	High; not available as a standardized commercial product	Limited to small single-center studies; no commercial standardization	Research stage
EasySep Direct/RosetteSep	Not established in cutaneous melanoma	Low; no dedicated instrument required	Limited enrichment specificity and absence of standardized enumeration approaches	Research stage
CTC-iChip	Not established	High; custom microfluidic setup; expert operation required	Technically demanding and not scalable beyond specialized research centers; no commercial standardization	Research stage
HB-CTC-Chip	Not established	High; custom setup	Single pilot cohort; no independent replication; not commercially available	Research stage
Diagnostic leukapheresis	Not standardized for CTC-specific application	High; apheresis unit and clinical setting required	Invasive procedure; not compatible with routine longitudinal monitoring	Research stage
PAFC (Cytophone)	Not established	Very high; specialized equipment required	No cell harvest possible; not applicable to low-melanin CTCs	Experimental
DEPArray (secondary platform only)	Reproducibility dependent on the primary enrichment platform	Very high; requires prior primary enrichment	Not a standalone CTC enrichment platform; research use only	Research stage

Among the available technologies, CellSearch with the melanoma-specific MCAM/MCSP kit is the most clinically advanced, supported by standardized protocols and prospective studies. However, the kit is not FDA-cleared for melanoma, and the proposed prognostic thresholds have not yet been validated across independent multicenter cohorts. ISET, ScreenCell, and Parsortix have also been evaluated in melanoma patients but still lack standardized protocols, regulatory approval for melanoma, or prospective multicenter validation. Most other approaches, including microfluidic, combined, and *in vivo* methods, remain at an experimental stage and have been assessed only in small cohorts or single-center studies.

A major limitation shared by almost all platforms is the lack of inter-laboratory reproducibility studies in cutaneous melanoma. This is particularly relevant because melanoma CTCs are highly heterogeneous, and no single enrichment strategy can capture the entire circulating tumor cell population. In addition, the non-epithelial origin of melanoma makes EpCAM-based approaches unsuitable. As a result, detection rates vary considerably between studies, and no standardized protocol or reporting criteria have been established. Despite encouraging prognostic data, the lack of standardization, prospective validation studies, and clinically validated thresholds currently limits the routine clinical use of CTC analysis in cutaneous melanoma.

## 5 Conclusion

Current platforms allow isolation and molecular characterization of melanoma CTCs, but none capture the full spectrum.

Future developments will likely focus on standardized, hybrid, and combinatorial approaches to improve recovery, purity, and molecular resolution. Longitudinal and molecular analyses can reveal treatment response, early progression, and relapse in real time, addressing key unmet clinical needs.

Nevertheless, several limitations currently hamper CTCs analyses in clinical practice. The rare frequency of CTC in bloodstream (1–10 CTCs in 8 mL of blood) and the low sensitivity of CTC detection methods represent a major challenge. The inability to capture all CTC can lead to false negative result and thus hindering their clinical value. In addition to the low number of CTC in blood, high phenotypic heterogeneity observed in melanoma CTCs adds significant complexity to the development of methods that can capture CTCs able to fully represent the entire tumor heterogeneity or tumor evolution over time reducing CTCs usefulness as biomarker of minimal residual disease (MRD) or response to treatment.

Although melanoma CTC is not a replacement for conventional diagnostics, their analysis has shown strong potential as prognostic and monitoring biomarker. The lack of standardized isolation and detection methods currently hinders its implementation in routine clinical practice.

For this reason, further methodological standardization and prospective validation studies are required before CTC based assays can be broadly adopted in clinical decision-making.

## Glossary

ABCB5: ATP Binding Cassette Subfamily B Member 5
CAF: Cancer-associated fibroblast
CDX: Cell line-derived xenograft
CK: Cytokeratin
CSC-CTC: Cancer stem cell-like CTC
CSPG4: Chondroitin sulfate proteoglycan 4
CTCs: Circulating tumor cells
ctDNA: Circulating tumor DNA
DAPI: 4′,6-diamidino-2-phenylindole
ddPCR: droplet digital PCR
DFS: Disease-free survival
EMT: Epithelial-mesenchymal transition
EpCAM: Epithelial cell adhesion molecule
EVs: Extracellular vesicles
FCM: Multimarker flow cytometry
FDA: Food and Drug Administration
FMSA: Flexible micro spring array
GEDI: Geometrically enhanced differential immunocapture
GO: Graphene oxide
gp100: Glycoprotein 100, PMEL
HB: Herringbone
ICC: Immunocytochemistry
ISET: Isolation by Size of Epithelial Tumor Cells
LCM: Laser capture microdissection
LDH: Serum lactate dehydrogenase
MCA: Microcavity array
MCAM: Melanoma cell adhesion molecule
MCSP/CSGP4/HMW-MAA: Melanoma-associated chondroitin sulfate proteoglycan
MGH: Massachusetts General Hospital
MLANA: Melan-A, MART-1
MRD: Minimal residual disease
MTNSU: Microchip-based high-throughput micro sampling unit
ncRNA: Non-coding RNA
OS: Overall survival
PA: Photoacoustic
PBMCs: Peripheral blood mononuclear cells
PDMS: Polydimethylsiloxane
PFS: Progression-free survival
RANK: Receptor activator of nuclear factor κ B
RT-qPCR: Reverse transcription quantitative PCR
RBCs: Red Blood Cells
RFS: Recurrence-free survival
RT-PCR: Reverse transcriptase-PCR
S100: S100 Calcium Binding Protein
SiNP: Silica nanoparticle
SLNB: Sentinel lymph node biopsy
TAC: Tetrameric antibody complex
TILs: Tumor-infiltrating lymphocytes
UVR: Ultraviolet radiation
VTX-1: Vortex
WBC: White blood cell
WGA: Whole genome amplification
